# JIAPS is Indexed in PubMed

**DOI:** 10.4103/0971-9261.59598

**Published:** 2009

**Authors:** K. L. N. Rao

**Affiliations:** Department of Pediatric Surgery, Postgraduate Institute of Medical Education and Research, Chandigarh – 160 012, India E-mail: klnrao@hotmail.com

Journal of Indian Association of Pediatric Surgeons is now indexed with PubMed. I proudly convey this message to members of the Indian Association of Pediatric Surgeons and other readers of JIAPS. This means that now one can search for articles published in JIAPS from 2008 onwards, through PubMed. The full texts of JIAPS articles are archived with PubMed Central (PMC) and searchable separately from Entrez PubMed.

We are already indexed with several important bibliographic databases as Bioline International, Hinari, Index Copernicus, IndMed, MedInd, Scimago Journal Ranking, SCOPUS, etc. but indexing in PubMed was a long pending issue with the journal in the past few years. Achieving this feat has taken a dedicated editorial team and the publisher who have strived hard towards the indexing goal.

The Journal was started in October 1995 by the then Editor-in-Chief, Prof. Subir K. Chatterjee. I put on record my appreciation of the services of various editorial teams, D. Basak, A.K. Basu, D.K. Gupta, Sandeep Agarwala and my colleague Ravi Kanojia for shouldering the responsibilities tirelessly. I would take this opportunity to thank all the other editorial team members and Medknow Publishers whose efforts have made this paradigm shift happen today with JIAPS.

With this achievement, we now hope that the journal will have an inflow of quality scientific work both from India and abroad. The onus of the quality of the journal still lies on the author who favors a particular journal; the editorial team is just a catalyst to improve the outcome. The journal now needs to scale greater heights and strive to achieve citations and impact factor. This can be done by publishing greater number of original articles. Unfortunately, at present, only 20% of the submitted manuscripts are original and review articles, and rest of the submissions are case reports. I would request all prospective authors, publishing papers in international journals just because they are indexed, to choose JIAPS over the others now, as we are now in equivalence with international journals as far as indexing is concerned.

Once again I congratulate all the authors, reviewers, editorial team members and the publisher for making this happen to JIAPS.

